# Structural and Functional Aspects of the Neurodevelopmental Gene *NR2F1*: From Animal Models to Human Pathology

**DOI:** 10.3389/fnmol.2021.767965

**Published:** 2021-12-15

**Authors:** Chiara Tocco, Michele Bertacchi, Michèle Studer

**Affiliations:** Université Côte d’Azur, CNRS, Inserm, iBV, Nice, France

**Keywords:** BBSOAS, neurodevelopmental disease, *NR2F1*, mouse models, cortical development

## Abstract

The assembly and maturation of the mammalian brain result from an intricate cascade of highly coordinated developmental events, such as cell proliferation, migration, and differentiation. Any impairment of this delicate multi-factorial process can lead to complex neurodevelopmental diseases, sharing common pathogenic mechanisms and molecular pathways resulting in multiple clinical signs. A recently described monogenic neurodevelopmental syndrome named Bosch-Boonstra-Schaaf Optic Atrophy Syndrome (BBSOAS) is caused by *NR2F1* haploinsufficiency. The *NR2F1* gene, coding for a transcriptional regulator belonging to the steroid/thyroid hormone receptor superfamily, is known to play key roles in several brain developmental processes, from proliferation and differentiation of neural progenitors to migration and identity acquisition of neocortical neurons. In a clinical context, the disruption of these cellular processes could underlie the pathogenesis of several symptoms affecting BBSOAS patients, such as intellectual disability, visual impairment, epilepsy, and autistic traits. In this review, we will introduce NR2F1 protein structure, molecular functioning, and expression profile in the developing mouse brain. Then, we will focus on Nr2f1 several functions during cortical development, from neocortical area and cell-type specification to maturation of network activity, hippocampal development governing learning behaviors, assembly of the visual system, and finally establishment of cortico-spinal descending tracts regulating motor execution. Whenever possible, we will link experimental findings in animal or cellular models to corresponding features of the human pathology. Finally, we will highlight some of the unresolved questions on the diverse functions played by Nr2f1 during brain development, in order to propose future research directions. All in all, we believe that understanding BBSOAS mechanisms will contribute to further unveiling pathophysiological mechanisms shared by several neurodevelopmental disorders and eventually lead to effective treatments.

## Introduction

Neurodevelopmental disorders (NDDs) are a highly heterogeneous class of mainly genetic pathological conditions, often due to defects of early mechanisms of brain development, such as cell proliferation, migration and differentiation, as well as activity and connectivity. Such an early origin represents a great challenge for scientists aiming at investigating physiological and pathological mechanisms underlying nervous system development. While historically *in vivo* mouse and *in vitro* two-dimensional (2D) cell culture models have been the favorite experimental approaches to study NDDs, new techniques such as three-dimensional (3D) organoids carrying patient-specific mutations have been recently developed and are now extensively coupled with more traditional methods.

The high heterogeneity of NDDs is reported at both genetic and clinical levels, with several causative genes and variable genotype-dependent severity of multiple clinical signs (Cristino et al., 2014; Hormozdiari et al., 2015; Parenti et al., 2020). However, despite such heterogeneity, NDD patients carrying distinct mutations often present a comorbidity of multiple symptoms [e.g., intellectual disability (ID), autism spectrum disorder (ASD), and epilepsy; van Bokhoven, 2011; Du et al., 2018; Parenti et al., 2020], suggesting the existence of common molecular pathways ultimately converging on similar clinical features. Recent insights in support of this hypothesis showed that several NDD-causative genes are involved in protein homeostasis, such as the mTOR pathway (Kelleher and Bear, 2008; Sahin and Sur, 2015; Borrie et al., 2017; Parenti et al., 2020), transcriptional and epigenetic regulation (Ronan et al., 2013; Parenti et al., 2020), and synaptic signaling and plasticity (Bourgeron, 2015; Südhof, 2018; Parenti et al., 2020). Hence, studying the molecular mechanisms of a specific syndrome might also help to shed light on NDDs in general, eventually leading to faster diagnosis and better treatments for patients.

Among NDDs, a recently described genetic condition called Bosch-Boonstra-Schaff Optic Atrophy Syndrome (BBSOAS) has been first reported in 2014 (Bosch et al., 2014). Till now, about 100 patients have been diagnosed (Rech et al., 2020), but more cases are regularly identified worldwide, indicating that the prevalence of this new syndrome is still, most probably, underestimated. BBSOAS symptoms are very heterogeneous and combine, among others, intellectual deficits, global developmental delay, epilepsy, motor dysfunctions and autistic traits, often associated with optic nerve atrophy and cerebral visual impairment. The combination of clear cognitive and visual disorders makes this syndrome quite unique and distinct from other NDDs.

BBSOAS is caused by *NR2F1* haploinsufficiency, implying that all patients so far identified carry a non-functional *NR2F1* allele, either due to deletion or missense point mutations that compromise its expression levels and/or its molecular activity. Although reported mainly as *de novo* mutations, a few BBSOAS inherited variants have been recently described (Rech et al., 2020; Jurkute et al., 2021). The BBSOAS causative gene -*NR2F1*- and its homolog *NR2F2* code for transcriptional regulators belonging to the superfamily of steroid/thyroid hormone receptors. Both factors are considered as orphan nuclear receptors, since their physiological ligands have not been identified yet (Wang et al., 1989, 1991; Qiu et al., 1995; Pereira et al., 2000). Their protein structure resembles that of other members of the family, with two highly conserved domains: a zinc-finger DNA binding domain (DBD) able to recognize target DNA sequences (direct repeats spaced by one nucleotide, called DR1), and a putative ligand binding domain (LBD), necessary for dimerization and binding of cofactors (Tsai and Tsai, 1997). Variable degrees of symptom severity have been reported among BBSOAS patients with distinct point mutations, suggesting the existence of a genotype-phenotype correlation. In particular, BBSOAS patients carrying *loss-of-function* mutations in the DBD display more severe clinical features compared to patients with variants in other regions of the protein (Rech et al., 2020). As NR2F1 binds the DNA in the form of dimers, this genotype-phenotype correlation could be due to a dominant negative effect of a mutated NR2F1 protein over a normally functional one during dimerization.

Thanks to the high degree of homology between human *NR2F1* and mouse *Nr2f1* orthologs, several mouse models have been employed to mimic BBSOAS pathogenesis and investigate the underlying neurodevelopmental processes. In this review, we will introduce the structure and molecular function of *Nr2f1* as a key transcriptional regulator orchestrating mouse brain development, able to both activate or repress the expression of target genes depending on the cellular context. We will then introduce a summary of the roles played by Nr2f1 in distinct developmental contexts: regulation of cortical neuron differentiation and establishment of area identity; modulation of intrinsic electric neural properties during corticogenesis; control of interneuron generation, hippocampal formation, sensorimotor system establishment and, finally, assembly of peripheral and central visual systems. Furthermore, we will relate some of the Nr2f1 functions, described in animal models, to corresponding symptoms reported in BBSOAS patients. Finally, we will discuss recently developed methodological approaches that could be employed to further unravel NR2F1 roles in both physiological and pathological brain development.

## *Nr2f1* Molecular Structure and Transcriptional Regulation Mechanisms

**Figure 1:**
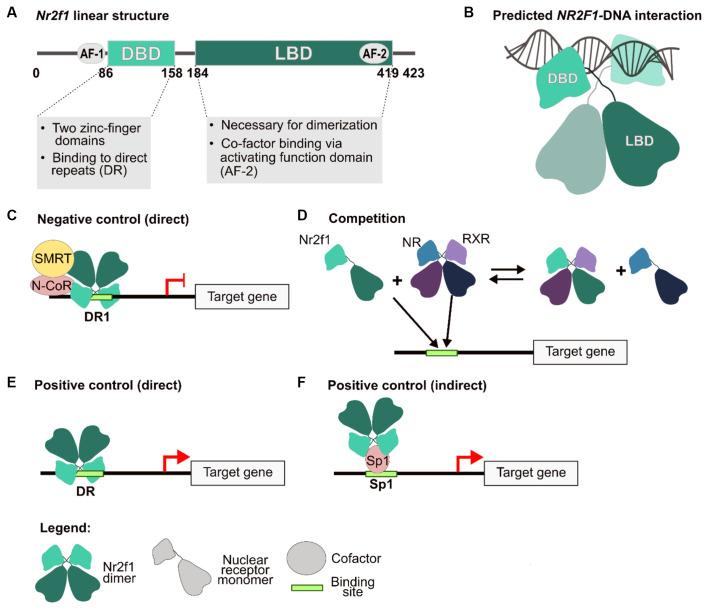
NR2F1 structure and molecular mechanisms of gene expression regulation. **(A)** Human NR2F1 linear structure showing conserved protein domains. The activation function (AF) 1 and 2, the DNA-binding domain (DBD) and ligand-binding domain (LBD) are depicted as circles and boxes, respectively. **(B)** Schematic model of an NR2F1 dimer, based upon predictive homology obtained by using NR2F2 structure as a template (96% aminoacidic sequence homology with NR2F1; Kruse et al., 2008). In the scheme, the LBD mediates the dimerization, whereas the DBD, consisting of two highly conserved zinc finger domains, are implicated in the interaction with the double DNA helix. Between the two domains, an undefined region depicted as a loose string, which does not contain secondary structures, has not been resolved to date. **(C–F)** Different regulatory mechanisms carried out by Nr2f1. The nuclear receptor can act both as a direct inhibitor **(C)**, as a competitor by binding to the regulatory sequence of target genes, and/or by sequestering other nuclear receptors as heterodimers **(D)**, or as an activator, either directly **(E)** or indirectly **(F)**, by interacting with other factors, such as Sp1, to induce transcription. AF-1/2, activation function domain 1 and 2; DBD, DNA-binding domain; DR1, direct repeat sites (spaced by one nucleotide); N-CoR, nuclear receptor co-repressor; NR, nuclear receptor; RXR, retinoic X receptor; SMRT, silencing mediator of retinoic acid and thyroid hormone receptor; Sp1, transcription factor specificity protein 1. Modified from Tang et al. (2015) and Bertacchi et al. (2019b).

Since 1999, a unified nomenclature system has renamed *COUP-TF* as “*NR2F*” for *Nuclear Receptor Subfamily 2* *Group F* of the steroid/thyroid hormone superfamily of nuclear receptors (Auwerx et al., 1999). Prior to that, these factors were known as *COUP-TF*s, for “*Chicken Ovalbumin Upstream Promoter Transcriptional Factors*”, reflecting their first reported role in regulating the chicken ovalbumin gene expression through direct binding to its promoter region (Pastorcic et al., 1986; Sagami et al., 1986). They are also defined as “orphan” receptors, since the identity of their physiological ligands is still elusive. Two major homologs of *Nr2f*s have been identified in vertebrates: *COUP-TFI*/*NR2F1* and *COUP-TFII*/*NR2F2* (Wang et al., 1989; Qiu et al., 1995). Their molecular structure resembles that of other nuclear receptors of the same family, encompassing two highly conserved domains: the DNA binding domain (DBD; consisting of two conserved Zinc-finger motifs), and the ligand-binding domain (LBD; Figures 1A,B). Based on their highly conserved sequence (Pastorcic et al., 1986), orthologs in mice, rats, *Xenopus*, chicken, hamster, *Drosophila*, *C. elegans*, zebrafish, and sea urchin have also been cloned and functionally characterized (reviewed in Alfano and Studer, 2013). The homology between human and mouse *NR2F* genes is very high (ranging from 95% to 100% amino acid sequence homology, depending on the protein regions; Qiu et al., 1995; Alfano and Studer, 2013), suggesting that their functions and targets might be conserved in both species. Another functionally relevant region is the C-terminal activation domain (named AF-2), whose active conformational state allows the binding of cofactors to the LBD and ultimately controls the transcriptional regulation of target genes (Germain et al., 2006). This active conformation state is generally reached *via* interactions with specific ligands, as for example the binding of retinoic acid (RA) and activation of the AF-2 domain of Retinoid X Receptor-α (RXRα; Bourguet et al., 1995; Egea et al., 2000; Germain et al., 2006). In the specific case of NR2F2, crystallographic studies have shown that the LBD is normally present in an auto-inhibited conformation, due to the binding between the AF-2 and cofactor binding sites, and that this auto-repressed state can be reverted with high concentration of RA (Kruse et al., 2008). Due to the high homology between NR2F1 and NR2F2, it is reasonable to speculate that a similar mechanism is also valid for NR2F1, but this has not been tested yet. Furthermore, RA-mediated activation of NR2F2 is itself still under debate, as: (i) the concentration of RA used in the study were above physiological levels and (ii) NR2F members might follow different mechanisms of activation, included being intrinsically active and able to regulate target genes regardless of the presence of any ligand, as previously shown for other members of the family (Wang et al., 2003). In addition, since NR2F2 affinity for RA is quite low, it is conceivable that other unidentified endogenous ligands do exist and activate NR2F nuclear receptors more efficiently.

Differently from the highly conserved AF-2 domain, the N-terminus activation domain (AF-1), necessary for co-factor recruitment, shows a lower degree of homology between NR2F members and other orthologs. This could imply that NR2F factors bind to similar cis-responding elements on the DNA, but then greatly differ in their molecular interactions with co-factors, an important aspect for acquiring cellular- and time-specific functions.

As transcriptional regulators, NR2F/Nr2f factors can both promote or inhibit gene expression through several distinct molecular and cellular mechanisms (Cooney et al., 1992; Leng et al., 1996; Alfano and Studer, 2013). For instance, repression can be elicited through direct binding of the target gene regulatory sequence together with Silencing Mediator for Retinoid or Thyroid-hormone receptors (SMRT) or Nuclear receptor Co-Repressor (N-CoR) co-factors (Hwung et al., 1988; Cooney et al., 1992; Montemayor et al., 2010; Figure 1C), or *via* indirect mechanisms by sequestering important proteins for the transcriptional machinery (Evans and Mangelsdorf, 2014; Figure 1D). As positive regulators, Nr2fs have been found to act either by directly binding the regulatory sequence of their target genes (Figure 1E), or as co-factors of other transcription factors (such as Sp1) in the context of chromatin complexes (Leng et al., 1996; Pipaón et al., 1999; Figure 1F).

In some instances, Nr2fs are reported to recruit chromatin remodeling co-activators (e.g., *CREB Binding Protein* -*CBP*- and *Steroid Receptor Coactivator-1* -*SRC1*-) and induce H3K9 acetylation, finally resulting in an open chromatin state which facilitates gene expression (Montemayor et al., 2010). Additionally, Nr2f1 can also help recruit DNA methyltransferases and actively assist chromatin demethylation (Gallais et al., 2007). However, an opposite mechanism has also been reported. For instance, in dormant cancerous cells (Sosa et al., 2014) or virally Ad12-infected human cells (Smirnov et al., 2000), *Nr2f1* mainly acts as a global chromatin repressor. This dual, contrasting Nr2f function might be accomplished *via* a ligand-activated conformational change, a common mechanism of action for nuclear receptors (Cooney et al., 2001; Weikum et al., 2018), or by interaction with distinct co-factors depending on the cellular context.

In summary, NR2F/Nr2f factors can either function as activators or repressors of target genes found in a chromatin permissive state in a time- and region-specific manner or alternatively, as chromatin remodellers themselves, by either facilitating or repressing acetylation or methylation, possibly depending on their conformation. However, more studies are needed to further comprehend their mechanistic function during the regulation of target gene expression.

## One Gene to Rule Them All: *Nr2f1*-Dependent Regulation of Cell Proliferation, Differentiation, and Migration

Due to a highly dynamic pattern of expression, the ability to interact with distinct sets of co-factors and the capacity to differentially regulate several target genes dissecting the cellular functions of transcription factors has always represented a challenging task. *Nr2f1* makes no exception as it plays multi-faceted, sometimes contrasting, roles during several developmental processes. As an example, *Nr2f1* can either positively or negatively regulate cell proliferation, differentiation, and migration, depending on the developmental time or co-expression of key co-factors. Despite this high level of complexity, several studies have started to unravel *Nr2f1* complex functions, especially in the context of neural progenitors and their progeny. At the cell-intrinsic level, *Nr2f1* can: (a) regulate cell cycle dynamics, in turn affecting the balance between neuronal progenitor proliferation and differentiation (Faedo et al., 2008; Bertacchi et al., 2020); (b) influence neuronal migration (Adam et al., 2000; Alfano et al., 2011; Touzot et al., 2016; Parisot et al., 2017), axonal elongation, and arborization (Qiu et al., 1997; Zhou et al., 1999; Armentano et al., 2006); (c) control identity and temporal competency of neuronal progenitor cells (Faedo et al., 2008; Naka et al., 2008; Okano and Temple, 2009); and (d) establish area-specific identity in progenitors and neurons (Zhou et al., 2001; Armentano et al., 2007; Tomassy et al., 2010; Alfano et al., 2014; Harb et al., 2016). In the following sections, we will summarize *Nr2f1* roles in these fundamental cellular processes, and the corresponding pathophysiological consequences that could result from *NR2F1* mutations in BBSOAS patients.

## *Nr2f1* Shapes Neocortical Morphology by Regional-Specific Modulation of Neurogenesis

**Figure 2:**
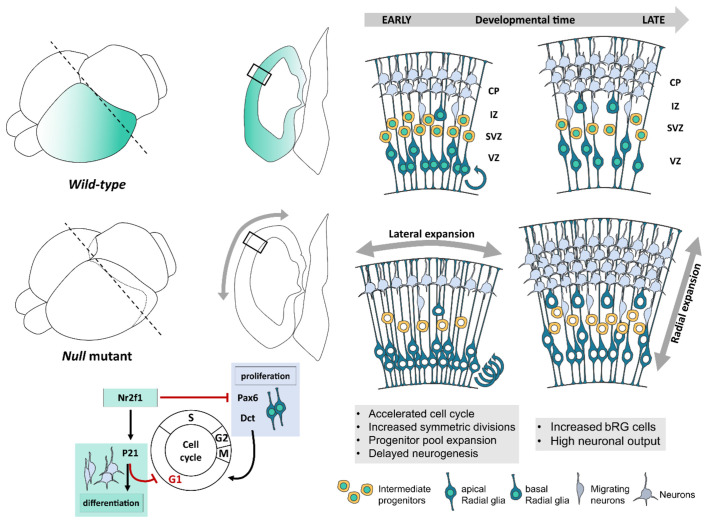
*Nr2f1*-mediated control of cellular and morphological dynamics during early corticogenesis. Schematic illustration of *Nr2f1* antero-low to postero-high expression gradient (blue color code, brain scheme on the left) and latero-high to medial-low gradient (transversal sections on the right) in the developing neocortex. *Nr2f1* expression in neural progenitors (NPs) spans from the ventricular zone (VZ) in apical radial glia cells (aRGs) to sub-ventricular zone (SVZ) in intermediate progenitors (IPs) and basal RGs (bRGs). RGs produce neurons directly or indirectly, *via* IPs, newly generated neurons migrating towards the intermediate zone (IZ) and then forming distinct layers in the cortical plate (CP). Upon *Nr2f1* loss (*null* mutants), the posterior NP pool expands leading to an occipital enlargement, reminiscent of megalencephaly. The NP pool expansion is caused by cell cycle acceleration (round arrows), increased symmetrical divisions and self-renewal, and delayed neurogenesis causing an early decrease of IPs and of migrating and differentiating neurons in IZ and CP. At later stages, IPs, bRGs and neurons are produced at a high rate, leading to the formation of a thicker posterior CP in mutant embryos compared to *wild type*. The morphological consequences of *Nr2f1* loss are an early lateral expansion followed by late radial expansion (gray arrows) of cortical hemispheres. At the molecular level, *Nr2f1* orchestrates NP cell cycle progression and neural differentiation by repressing Pax6 and Dct and thus cell cycling progression, while activating P21-mediated cell cycle exit to promote terminal neural differentiation. aRG, apical radial glia; bRG, basal radial glia; CP, cortical plate; IZ, intermediate zone; SVZ, sub-ventricular zone; VZ, ventricular zone.

The formation of the neocortex, the most highly evolved part of the mammalian brain, starts early during embryogenesis when neural progenitors (NPs) expand by proliferating, and then differentiate into all distinct neuronal subpopulations of the adult brain. In the forming mouse neocortex, early NPs called apical radial glia (aRG) cells, produce cortical neurons in a direct or indirect way, *via* intermediate progenitors (IPs; Figure 2). Then, newly generated neurons migrate to the cortical plate (CP), creating layers of radially organized neuronal classes that extend their axons to form brain circuits. In recent years, additional subtypes of self-renewing RG cells have been described in the developing human cortex, including basal radial glia (bRG; Hansen et al., 2010; Nonaka-Kinoshita et al., 2013; Pilz et al., 2013; Pollen et al., 2015). Notably, bRG cells are responsible for the abundant production of upper layer neurons in humans and other primates (Pollen et al., 2015; Nowakowski et al., 2016), and ultimately concur to the expansion of the cortical surface and the formation of neocortical convolutions. The gyrification, i.e., the folding of the cortical surface that generates convolutions (gyri) separated by spaces (sulci), provides an increased surface of the neuronal tissue to fit the intracranial space. Despite the significance of gyrencephaly and its link with brain size still being debated (Kelava et al., 2013; Zilles et al., 2013), evidence from several studies shows a strong correlation between cortical morphology and gyrification defects, and the onset of neurodevelopmental diseases (Casanova et al., 2004; Lin et al., 2007; Wolosin et al., 2009; Zhang et al., 2010; Lebed et al., 2013; Juric-Sekhar and Hevner, 2019).

Interestingly, some BBSOAS patients show specific neocortical malformations, such as macrocephaly and ventricular enlargement/asymmetry (Chen et al., 2016; Kaiwar et al., 2017), suggesting that impairments in the basic mechanism of NP self-renewal and neurogenesis could be associated with *NR2F1* haploinsufficiency. The recent characterization of the cortical morphology by Magnetic Resonance Imaging (MRI) in six novel patients has unraveled aberrant convolutions in the form of polymicrogyria-like brain malformations, or dysgyria, in the temporo-parieto-occipital (TPO) cortex, a brain territory heavily involved in several high-level neurological functions (Bertacchi et al., 2020). These cortical defects hint at a new and distinct role for the human *NR2F1* gene in controlling gyrification. The presence of cortical malformations in BBSOAS patients links the *NR2F1* gene to the heterogeneous group of neurodevelopmental diseases, called malformations of cortical development (MCD), in which structural brain anomalies and abnormal gyrification are associated with syndromic features, such as mild to moderate ID, infantile spasms and impaired oromotor skills (Jansen and Andermann, 2005; Manzini and Walsh, 2011; Barkovich et al., 2012; Guerrini and Dobyns, 2014; Parrini et al., 2016; Juric-Sekhar and Hevner, 2019). Notably, folding defects observed in BBSOAS patients affect the supramarginal and angular gyri, regions known to be involved in various aspects of language and emotional responses, memory retrieval, attention, and number processing (Stoeckel et al., 2009; Seghier, 2013; Oberhuber et al., 2016), suggesting that such malformations could be linked to the reported cognitive deficits.

In humans as in mice, the balance between self-renewal (proliferation) and neurogenesis (neuronal differentiation) of cortical NP cells needs to be tightly regulated, and their radial migration as well as laminar organization regionally controlled, in order to properly shape the mature cerebral cortex (Florio and Huttner, 2014; Villalba et al., 2021). An imbalance between self-renewal and neurogenesis impairs the production of the number and type of neurons during corticogenesis; as an example, excessive early neurogenesis will deplete the progenitor pool and result in a microcephalic brain with fewer neurons, whereas disproportionate proliferation might delay neurogenesis and produce a macrencephalic brain. Several determinant genes are involved in this process, including *Nr2f1*.

The use of an *Nr2f1* constitutive (*null*) mutant mouse as a BBSOAS model showed that *Nr2f1* can finely regulate the self-renewal/differentiation balance of specific neocortical progenitor classes. Differently from other NDD mouse models, this control turned to be regionalized, in accordance with *Nr2f1* antero-posterior graded expression displaying highest levels in the occipital cortex in mice (Liu et al., 2000; Zhou et al., 2001; Armentano et al., 2006; Tomassy et al., 2010), and in humans (Alzu’Bi et al., 2017; Bertacchi et al., 2020; Foglio et al., 2021). In the caudal cortex, *Nr2f1* promotes asymmetric divisions by driving NP differentiation (Faedo et al., 2008; Bertacchi et al., 2020); hence, *Nr2f1* loss resulted in delayed neurogenesis and amplification of both apical and basal RG cells, ultimately leading to expanded occipital hemispheres (Bertacchi et al., 2020; Figure 2). Furthermore, bRGs, which are normally scarcely represented in murine brains (Reillo et al., 2011; Wang et al., 2011), are abnormally enlarged in *Nr2f1* mutant brains (Figure 2). An increase of the progenitor pool is also in line with a slight increase of the overall neocortical volume measured by MRI in adult *heterozygous* *(HET)* mice (Chen et al., 2020) and consistent with expanded/elongated occipital convolutions observed in two BBSOAS patients (Bertacchi et al., 2020). At the molecular level, *Paired box protein-6* (*Pax6*) could be partially responsible for the caudal increase of progenitors and neurons (Faedo et al., 2008; Bertacchi et al., 2020), being directly regulated by *Nr2f1* (Tang et al., 2010). *Nr2f1* mutants showed sustained levels of Pax6 in RG cells, in particular caudally, where Pax6 is normally expressed at low levels (Bertacchi et al., 2020), and known to modulate bRG amplification (Wong et al., 2015). Similarly to *Nr2f1*, mouse *Pax6* is a master regulator of NP cell cycle, differentiation rate and, more in general, neocortical development and area mapping (Bishop et al., 2000, 2002; Estivill-Torrus et al., 2002; Englund et al., 2005; Asami et al., 2011). Consistently, mutations in the human *PAX6* gene are also associated with MCDs and polymicrogyria-like malformations (Mitchell et al., 2003; Spalice et al., 2009).

Together with *Nr2f1* and *Pax6*, other key master genes of neocortical development, such as *Empty spiracle homeobox-2* (*Emx2*), *Specificity Protein-8* (*Sp8*) and *Forkhead box protein G1* (*Foxg1*), operate a similar control of cell cycle progression, NP proliferation, neurogenesis and neuronal maturation (Bishop et al., 2000; Martynoga et al., 2005; Samson et al., 2005; Dehay and Kennedy, 2007; Zembrzycki et al., 2007; Georgala et al., 2011; Mi et al., 2013; Borello et al., 2014; Borrell and Calegari, 2014), even though only a few of them act in a regionalized manner. Hence, *Nr2f1* could belong to a wider gene network orchestrating cortical development along the antero-posterior (A-P), dorso-ventral (D-V), and latero-medial (L-M) cortical axes (Alfano and Studer, 2013; Bertacchi et al., 2019b; Cadwell et al., 2019). A dose-dependent combinatorial code of these and other genes could prompt NPs to acquire different neurogenic potentials depending on their spatial coordinates, ultimately operating an area-specific control of the number and type of neurons locally produced in distinct cortical regions (Bertacchi et al., 2019b).

However, while mouse models greatly advance our knowledge on *Nr2f1*-dependent control of NP physiology, it is useful to remember that, contrary to humans and primates, the mouse brain has a smooth surface without any convolution (namely “lissencephalic”) and contains a very small population of bRG cells; hence, murine models are not optimal to challenge *Nr2f1* and similar genes in bRG-dependent processes of cortical convolution and gyrus morphology. Further experiments in gyrencephalic experimental models, such as the ferret or the marmoset (Kelava et al., 2013), will be necessary to assess *Nr2f1* contribution to gyrencephaly and better correlate experimental data to BBSOAS clinical observations.

## *Nr2f1* Regionally Controls Cell Cycle Dynamics of Neocortical Progenitors

One of the mechanisms underlying the balance between NP proliferation and neurogenesis is the tight control of cell cycle progression, either in terms of checkpoint regulation or duration of the distinct phases (Dehay and Kennedy, 2007; Borrell and Calegari, 2014). These steps are coordinated by genes directly involved in cell cycle dynamics but also by transcription factors acting upstream of cell-cycle genes.

A recent study shows that *Nr2f1* directly controls cell cycle length, particularly in the caudal cortex by acting as a break. Indeed, *Nr2f1*-loss results in G1-phase shortening and overall cell cycle acceleration, finally leading to progenitor pool amplification and possibly impacting cortical area size (Bertacchi et al., 2020; Figure 2). The extension of cortical areas and the size of their neuronal pool had been previously shown to be affected by cell cycle duration and rate of NP cell cycle re-entry (Lukaszewicz et al., 2005). In fact, enlarged brain size can be recreated by artificially accelerating cell cycle progression (Nonaka-Kinoshita et al., 2013). NPs with shorter cell cycle duration, due to a significantly shorter G1-phase, have been demonstrated to be “younger” and to favor self-renewal over neurogenesis (Calegari et al., 2005; Lange et al., 2009; Arai et al., 2011). Hence, the accelerated cell cycle in *Nr2f1* mutants, associated with delayed neurogenesis, suggests that loss of *Nr2f1* brings back the biological clock of NPs and that Nr2f1 acts as a temporal regulator of corticogenesis, as previously suggested (Naka et al., 2008). This is partially mediated by the cyclin-dependent kinase inhibitor P21 (Bertacchi et al., 2020), which triggers cell cycle exit and differentiation in NPs (Siegenthaler and Miller, 2005; Buttitta and Edgar, 2007; Heldring et al., 2013; Figure 2). However, the role of *Nr2f1* in the G1-to-S phase transition and thus in cell cycle regulation is strictly tissue specific. Indeed, while mouse *Nr2f1* promotes cell cycle exit also in medial and caudal ganglionic eminences (Lodato et al., 2011; Touzot et al., 2016), as in the neocortex, it instead promotes cell cycle progression in other brain regions, such as the hippocampus (Parisot et al., 2017). Hence, further studies will be necessary to establish how tissue-specific co-factors contribute to redirecting *Nr2f1* function towards either activating or inhibiting cell cycle progression.

## *Nr2f1*-Mediated Regulation of Neuronal Migration in Several Cell Types

In addition to defective NP cell cycle progression, abnormal migration of newly differentiated neurons can further impact cortical morphology and layer organization of the nascent cortical plate, converging to cause specific features of NDDs. As an example, altered neuronal migration can impair cortical layering, resulting in ectopic nodular heterotopia (clusters of neurons stuck in ectopic position; Guerrini and Parrini, 2010; Guerrini and Dobyns, 2014; Watrin et al., 2015). Such morphological defects often correlate with ID and possibly concur to trigger epileptic traits (Aghakhani et al., 2005). *Nr2f1* has been proved to control neuronal migration first in cell cultures (Adam et al., 2000), and then in both embryonic (Alfano et al., 2011; Lodato et al., 2011; Parisot et al., 2017) and postnatal stages of brain development (Bovetti et al., 2013; Flore et al., 2016; Bonzano et al., 2018). In migrating neurons, *Nr2f1* transcriptionally controls the expression of the *Rho family GTPase-2* (*Rnd2*) known to act on actin cytoskeleton organization (Azzarelli et al., 2015). Upon* Nr2f1* loss, *Rnd2* levels are highly increased, impairing the bipolar-to-multipolar state transition of late-born migrating cortical neurons and thus affecting their laminar localization (Alfano et al., 2011; Bertacchi et al., 2019b). Moreover, *Nr2f1* loss leads to reduced dendritic arborization and axonal defects of cortical upper layer neurons (Alfano et al., 2011). Together, these morphological defects might explain the thinning of the corpus callosum described in mutant mice (Armentano et al., 2006; Alfano et al., 2011) and reported in BBSOAS patients (Bosch et al., 2014; Chen et al., 2016; Rech et al., 2020). Impaired differentiation and/or migration of other projection neurons most probably impact the formation of further brain commissures and long-range tracts, as previously described (Armentano et al., 2006). Consistently with *Nr2f1* role in controlling migration, some *NR2F1*-haploinsufficient patients have signs of periventricular neuronal heterotopia in their posterior cortex (Guerrini et al., 2009; Bertacchi et al., 2020). However, a direct causative link between *NR2F1* deletion/mutation and heterotopia formation is still missing to date.

## The Blueprint of Neocortical Organization: *Nr2f1* Graded Expression and Its Implication in Arealization

Besides being produced in the right number and at the correct developmental time, cortical neurons also need to adopt a specific identity along all cortical axes. The mechanisms which pattern the neocortex into distinct functional areas along its tangential surface are termed “arealization”, and imply a specialization of the generic six-layered structure to acquire distinct cytoarchitectures and peculiar abundance of several neuronal classes, reflecting individual area-specific functionality and wiring (O’Leary and Nakagawa, 2002; O’Leary and Sahara, 2008; Tomassy et al., 2010). As an example, the subpopulation of layer V corticofugal neurons, that send output information to subcerebral structures, such as the spinal cord and pontine nuclei, is broader in the adult primary motor area (M1) than in the primary somatosensory area (S1). Conversely, layer IV granular neurons, that receive sensory inputs from the thalamus, show the exact opposite trend. How cortical layers develop this regional diversity has been the subject of decades of research. Although the process is not yet completely elucidated, it appears to occur in two major steps: (i) the definition of an early protomap through the combinatorial graded expression of patterning genes, followed by (ii) an activity-dependent refinement of cortical area functionality (Cadwell et al., 2019).

**Figure 3:**
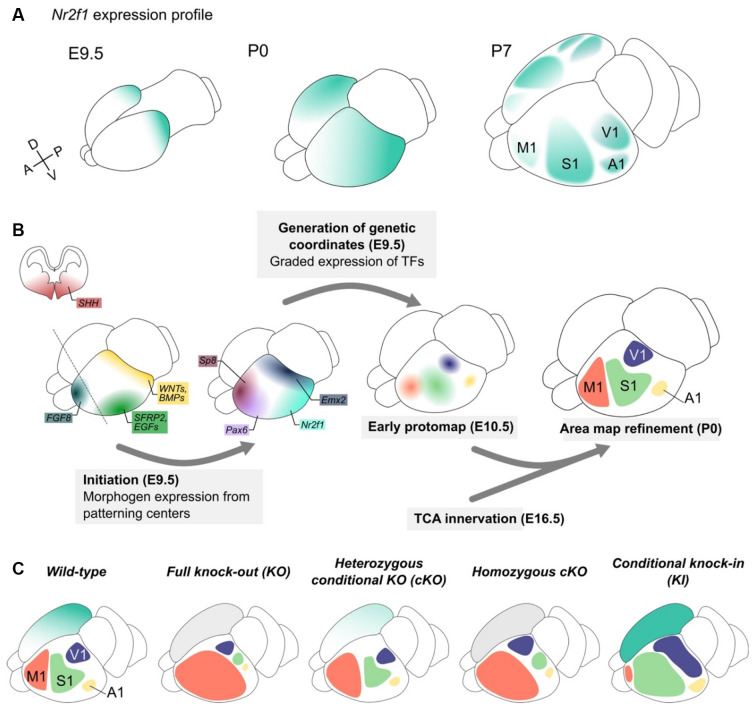
*Nr2f1* graded expression drives cortical arealization during embryonic development. **(A)** *Nr2f1* graded expression (blue color code) in the telencephalon starts at embryonic day (E) 9.5 posteriorly and then spreads forming a diffuse gradient from high postero-lateral to low antero-medial levels in the post-natal (P) 0 cortex. From the onset of area refinement (here represented at P7), *Nr2f1* high expression is maintained in the primary sensory areas (S1, V1 and A1), but almost absent from secondary sensory areas and from the frontal motor area (M1). **(B)** Graphic representing the different steps of the arealization process. Starting from E8.0, several morphogens (*SHH, WNTs, BMPs, SFRP2, EGFs, and FGF8* among others) are secreted from distinctly located patterning centers and diffuse along gradients in the developing neocortex, in turns driving the graded expression of transcription factors (TFs) in cortical progenitor cells. The strong caudal expression of *Emx2* and *Nr2f1* promotes the specification of sensory areas, while rostral high expression of *Pax6* and *Sp8* drives the specification of motor identity. This “protomap” is then refined with the arrival of thalamic axons (TCA innervation) conveying external sensory information. **(C)** Schematic of M1 (pink), S1 (green), V1 (purple) and A1 (yellow) areas in *wild-type* brains and in different mouse models of *Nr2f1* downregulation (constitutive knock-out -*KO-* and conditional one -*cKO-*) and upregulation (knock-in -*KI-*). The right hemisphere depicts *Nr2f1* expression, while the left one schematizes the position and size of distinct areas upon genetic manipulation. To note, changes in size and extension of the functional areas are not associated with significant changes in overall cortex volume, with the only exception of caudal megalencephaly in *null* animals (see Figure 2). A1, primary auditory area; M1, primary motor area; S1, primary somatosensory area; V1, primary visual area.

The characteristic *Nr2f1* graded expression in the cortical primordium and its maintenance in primary sensory areas advocate for its implication in early and late events of arealization (Zhou et al., 1999; Liu et al., 2000). In the mouse, *Nr2f1* expression starts at mouse embryonic (E) age 9.0–9.5 in the neuroectoderm, when the anterior neural plate starts to close (Qiu et al., 1994; Armentano et al., 2006). Then, its graded expression expands in several regions of the telencephalon, such as the cerebral cortex, hippocampus, thalamus, ganglionic eminences, and preoptic area (Armentano et al., 2006). In neocortical progenitors, its expression is higher in caudo-lateral and lower in rostro-medial regions, a gradient that is maintained in postmitotic cells and postnatally when areas are well-defined (Liu et al., 2000; Zhou et al., 2001; Armentano et al., 2007; Figure 3A).

Initial spatial coordinates are set up by morphogens, such as *Fibroblast Growth Factors* (*FGFs*), *Sonic Hedgehog* (*SHH*), *Retinoic acid* (RA), and *Bone Morphogenetic Proteins* (*BMPs*). These molecules are produced by signaling centers (O’Leary and Nakagawa, 2002; Shimogori et al., 2004; Samson et al., 2005; Shimogori and Grove, 2005) and are important for the establishment of proper coordinates along the A-P and D-V axes (Fukuchi-Shimogori and Grove, 2001; Grove and Fukuchi-Shimogori, 2003; Sur and Rubenstein, 2005; Sansom and Livesey, 2009; Greig et al., 2013) by acting in a dose-, context- and time-dependent manner (Figure 3B-initiation). Their main effectors are area patterning genes such as *Pax6, Sp8*, and *Emx2* among others, and *Nr2f1* itself (Grove and Fukuchi-Shimogori, 2003; O’Leary and Sahara, 2008; Alfano and Studer, 2013; Figure 3B-generation of genetic coordinates). Their combinatorial expression in neocortical progenitors provides precise spatial coordinates and regulates cell differentiation, area identity, neuronal maturation and network connectivity and function. Among early morphogens, *FGF8* plays a major role in the arealization process, by inducing rostrally-determining genes and repressing *Nr2f1* and other caudal genes (Garel et al., 2003; Samson et al., 2005; Storm et al., 2006; Toyoda et al., 2010). *Vice versa*, *Nr2f1* inhibits FGF signaling (but not *FGF8* expression) and antagonizes the expression of the major FGF8-activated gene, *Sp8* (Sahara et al., 2007; Faedo et al., 2008; Borello et al., 2014). However, expression gradients of area patterning genes in progenitors and early differentiating neurons only provide a pre-identity signature to the different proto-areas, with still no clear-cut boundaries and functions (Greig et al., 2013). Only a later activity-dependent refinement, conveyed by thalamocortical axonal afferences (TCA), leads to fully-shaped cortical areas with a clear distinction between primary and secondary functional areas (Figure 3B-TCA innervation).

## *Nr2f1* Specifies The Identity of Posterior Sensory Areas

A key role for *Nr2f1* in area mapping was first reported in a constitutive *Nr2f*1 k*nock-out* (named *KO* or *null*) mouse line, which displayed reduced expression of sensory cortical markers and enlarged expression of motor markers, with no clear cortical area subdivision (Zhou et al., 2001). In addition, TCAs failed to innervate layer IV in *null* animals, causing the premature death of these neurons and thus raising the possibility that impaired area division could just be an indirect consequence of the lack of thalamic inputs (Zhou et al., 2001). However, due to severe feeding problems, most *null* animals died perinatally (Qiu et al., 1997), hindering the study of cortical arealization at postnatal stages.

The use of cortico-specific *Nr2f1* *conditional KO (cKO)* mouse lines, in which *Nr2f1* expression was abolished either in cortical progenitor cells at E10.5 (Armentano et al., 2007) or specifically in postmitotic neurons at E11.5–E12 (Alfano et al., 2014) prevented any influence from the thalamus, as well as perinatal mortality, and thus allowed to elucidate the cortex-specific* Nr2f1* role during arealization. Although the neocortex of *Nr2f1* *KO* mutants was first described as a uniform “area-less” cortex (Zhou et al., 2001; O’Leary and Sahara, 2008), the analysis of both *cKO* mutants revealed that all sensory areas were still present, but greatly reduced in size (Armentano et al., 2007). While leaving the overall cortical surface unaffected, the cortex-specific ablation of *Nr2f1* influenced the size and location of the main functional areas, as demonstrated by several regionalized molecular markers and improper thalamo-cortical topography. For instance, primary somatosensory (S1), visual and auditory areas were all caudally misplaced and their size drastically reduced in mutants, in favor of an enlarged frontal motor (M1) area (Armentano et al., 2007). Because of the similar expression patterns observed between normal M1 and mutant S1, the area was therefore named “motorized S1” (mS1; Armentano et al., 2007; Tomassy et al., 2010). Although shifted, the relative position of adjacent areas was maintained upon loss of *Nr2f1*, as proven by the existence of a miniaturized S1 barrel field retaining normal genetic identity and TCA connectivity (Armentano et al., 2007; Figure 3C).

The cortical area defects observed in *Nr2f1* mutant brains (Armentano et al., 2007) are very severe compared to those resulting from the *loss-of-function* of other area patterning genes expressed solely in progenitors (Bishop et al., 2000, 2002; Mallamaci et al., 2000; Hamasaki et al., 2004; Manuel et al., 2007; Sahara et al., 2007; Zembrzycki et al., 2007, 2013; Tran et al., 2009). Furthermore, other genes belonging to the FGF pathway (i.e., FGF8 and Sp8) act in strong connection with *Nr2f1* and partially influence area patterning (O’Leary et al., 2007; Faedo et al., 2008, 2010; Borello et al., 2014). It is thus conceivable that FGF signaling and *Nr2f1* might act together as upstream regulators of a cascade of molecular events governing area patterning, whereas other genes such as *Pax6* and *Emx2* would act either as secondary downstream effectors or in parallel pathways and have a weaker effect on arealization. Finally, several chromatin regulators, such as the methyl-transferase *Setd2* (Xu et al., 2021), the transcription co-regulator *Cited2* (Fame et al., 2016; Wagner and MacDonald, 2021) and the epigenetic co-factor *LIM Domain Only-4* (*Lmo4*; Harb et al., 2016), have been reported to influence area identity refinement. Considering *Nr2f1* ability to recruit epigenetic factors (Montemayor et al., 2010), it might be interesting to investigate whether these or similar chromatin regulators act in association with or under the control of *Nr2f1* in the late arealization process.

In summary, the use of cortical conditional *KO (cKO)* models, compared to full *KO*s, has demonstrated that early area map impairments arise despite normal *Nr2f1* expression in thalamic neurons, pointing to an *Nr2f1* cell-intrinsic neocortical control of area identity (Armentano et al., 2007). Further experiments will be necessary to establish whether any area shift in *HET* animals, more similar to BBSOAS patients, has specific consequences on cortical function. Furthermore, whether *NR2F1* haploinsufficiency has consequences on human arealization, which could correlate with ID, is still an open question. Since a similar neocortical *NR2F1* gradient has been reported in human brains (Zhou et al., 2001; Armentano et al., 2007; Alzu’Bi et al., 2017; Molnár et al., 2019; Foglio et al., 2021), an evolutionarily conserved role for human *NR2F1* in area patterning is conceivable (Clowry et al., 2018).

## Post-Mitotic *Nr2f1* Expression Is Necessary and Sufficient for Neocortical Arealization

Initially established in progenitors, *Nr2f1* graded expression is maintained by post-mitotic neurons radially migrating into the cortical plate. *Nr2f1* inactivation solely in cortical post-mitotic cells reproduces the severe area defect obtained upon loss in progenitor cells, indicating that *Nr2f1* expression only in progenitors is not sufficient to maintain proper neuronal specification (Alfano et al., 2014; Bertacchi et al., 2019b). Conversely, *Nr2f1* overexpression in post-mitotic cells in a constitutive *Nr2f1 KO* model rescues sensory identity, laminar specification and topographic thalamocortical connectivity in S1 (Alfano et al., 2014). Hence, *Nr2f1* post-mitotic expression is necessary and sufficient to specify sensory (caudal) area identity in the developing neocortex. Similar mechanisms for the consolidation of neocortical arealization have started to emerge for other post-mitotic genes, such as *LIM homeobox-2* (*Lhx2*; Zembrzycki et al., 2015), *Pre-B-Cell Leukaemia Homeobox-1* (*Pbx1*; Golonzhka et al., 2015), *Basic Helix-Loop-Helix Protein-5* (*Bhlhb5*; Joshi et al., 2008), *T-Box Brain Transcription Factor-1* (*Tbr1*; Bedogni et al., 2010) and *COUP-TF interacting protein-1* (*Ctip1*; Greig et al., 2016). This indicates that genes expressed in young post-mitotic neurons play a fundamental role in neuronal and area specification, independently from progenitor identity (Fishell and Hanashima, 2008; Joshi et al., 2008; Bedogni et al., 2010; Greig et al., 2013).

The expression in both progenitors and neurons is a peculiar characteristic of *Nr2f1*, as other area patterning genes are expressed only in apical progenitors (*Pax6, Emx2*), or transiently in intermediate progenitors (*Tbr2, Ap2y*) or exclusively in post-mitotic cells (*Tbr1, Lhx2, Pbx1*). The preserved *Nr2f1* expression gradient in both cycling and differentiating cells could explain the strong effect on the area size and position observed in mutants, compared to that of other patterning genes. In this scenario, *Nr2f1* post-mitotic expression further reinforces areal identity specification initially imparted in progenitors (Alfano et al., 2014).

**Figure 4:**
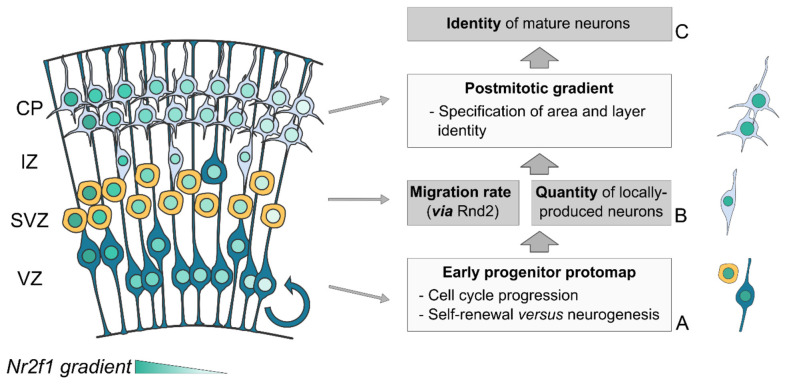
Triple *Nr2f1*-mediated control of neocortical mapping. The *Nr2f1* gradient in the developing neocortex (blue color code) is key for a regional-specific control of three fundamental processes of corticogenesis: cell proliferation **(A)**, cell migration **(B)** and neuronal identity acquisition **(C)**. **(A)** In progenitor cells, *Nr2f1* regulates cell cycle progression and the balance between self-renewal and differentiation (neurogenesis), ultimately controlling the number of newborn neurons locally produced along neocortical axes. **(B)** In newborn neurons, *Nr2f1* controls the rate and efficiency of radial migration , *via* the regulation of *Rnd2* expression. **(C)** Post-mitotically, *Nr2f1* leads the establishment and consolidation of mature neuron identity. Being expressed in both progenitors and neurons allows *Nr2f1* to connect all these distinct cellular processes, ultimately controlling the position and size of neocortical areas. CP, cortical plate; IZ, intermediate zone; SVZ, sub-ventricular zone; VZ, ventricular zone.

In summary, *Nr2f1* plays multiple roles during corticogenesis, mainly depending on the cellular context and developmental time (Figure 4). In cycling neural progenitors, it regulates cell cycle speed and balance between self-renewal and neurogenesis in a region-specific manner, ultimately determining how many neurons are produced (Faedo et al., 2008; Bertacchi et al., 2020). Notably, young neurons exiting the cell cycle radially migrate to the CP, and such migration rate is also regionally controlled by *Nr2f1* by directly regulating *Rnd2* expression (Alfano et al., 2011). Finally, *Nr2f1* influence over area identity is not limited to the establishment of an early progenitor protomap (Armentano et al., 2007; O’Leary et al., 2007; O’Leary and Sahara, 2008), but is most prominently accomplished *via* its post-mitotic role in the specification and consolidation of area and layer identity (Alfano et al., 2014). Being expressed in a gradient in both progenitors and neurons, *Nr2f1* can link different processes, ultimately orchestrating the number and type of neurons produced in a region-specific manner.

## Thalamic *Nr2f1* Expression Orchestrates Activity-Dependent Refinement of Cortical Areas

After the arealization process initiates prenatally, a further refinement is controlled postnatally, when TCAs start innervating the neocortex. In fact, the establishment of thalamocortical connectivity, responsible for relaying sensory input to their respective cortical targets, concours in refining boundaries between neocortical functional areas (Rubenstein and Rakic, 1999; Sur and Rubenstein, 2005; Rakic et al., 2009; Alfano and Studer, 2013). *Nr2f1* expression in sensory thalamic progenitors and neurons peaks at mid-gestation (Armentano et al., 2006; Zembrzycki et al., 2013), but is maintained at perinatal stages only in the lateral geniculate and ventro-posterior nuclei of the dorsal thalamus (Armentano et al., 2006).

The use of cortical *cKO* models showed that thalamo-cortical axons correctly reached the subplate at E16.5–E18.5, but that only a few succeeded in innervating the CP, while the majority were aberrantly wired. Additionally, cortico-thalamic projections were affected too. In normal conditions, projections from M1, S1 and V1 principally innervate the ventrolateral (VL), the ventroposterior (VP), and the dorsolateral genuculate (dLGN) nuclei of the thalamus, respectively. However, in cortical *cKO* brains, projections from both M1 and mS1 target the VL nucleus, with caudally displaced S1 projections connecting with the dLGN and very few projections reaching the VP (Armentano et al., 2007). Conversely, the use of thalamic *cKO* models showed that the genetic manipulation of sensory thalamic nuclei was sufficient to affect the organization of primary and secondary sensory areas in the neocortex (Chou et al., 2013; Vue et al., 2013; Antón-Bolaños et al., 2018). Accordingly, the selective inactivation of *Nr2f1* in the dLGN influences the organization of primary *versus* high-order (secondary) visual cortical regions, with V1 resulting virtually absent, in favor of enlarged higher-order areas (Chou et al., 2013).

In summary, the use of tissue-specific conditional mouse models has pointed to a dual role of *Nr2f1* during area mapping and formation: (i) cell-intrinsic specification of a proto-sensory neocortical map (*via* cortical intrinsic genetic programs and in tight link with FGF signaling); and (ii) non-cell-autonomous refinement of primary and secondary cortical maps through TCA inputs to their topographic cortical targets.

## *Nr2f1* Regulates Cell Intrinsic Electrophysiological Properties During Neocortical Maturation

Along with acquiring a precise cytoarchitectural organization, the mammalian neocortex is also characterized by the onset of spontaneous activity, an essential feature in specifying the composition and organization of neural circuits within and between functional areas (Jabaudon, 2017; Simi and Studer, 2018). The formation of an efficient network relies on several processes, including cell-intrinsic mechanisms regulating cell excitability of cortical pyramidal neurons, non-cell-intrinsic and activity-dependent fine-tuning *via* TCA innervation, as well as the correct balance between excitatory and inhibitory neuronal populations in mature circuits. Before a sensory-driven activity is conveyed to the cerebral cortex by TCAs, the developing cortex is already genetically primed to establish patterns of local spontaneous activity, characterized by large groups of synchronously firing neurons, and contributing to the generation of local neuronal circuits (Kirischuk et al., 2017; Andreae and Burrone, 2018; Antón-Bolaños et al., 2018; Luhmann and Khazipov, 2018). Cell-intrinsic regulation of excitability in neocortical neurons influences the formation of a functional somatotopic map, ultimately preparing cortical areas and circuits for upcoming sensory inputs (Antón-Bolaños et al., 2019). Simultaneously, spontaneous neuronal activity arises in the embryonic thalamus and is then conveyed to the immature cortex. As early patterns of spontaneous activity play a key role in setting the nascent cortical network organization, their alteration during cortical development leads to cortical circuit dysfunction (Kirkby et al., 2013; Li et al., 2013).

The impact of* Nr2f1* loss on neocortical arealization and neuronal differentiation raised the possibility that they could correlate with altered spontaneous activity. Other transcriptional determinants of cortical specification, such as *matrix metallopeptidase-9* (*MMP-9*) and *leucine-rich glioma inactivated-1* (*LGI-1*) factors (Boillot et al., 2016; Murase et al., 2016), have been shown to impinge on spontaneous activity. As another example, the transcription factor *Tbr1*, known to regulate cortical layer identity, also influences the intrinsic excitability of neocortical neurons (Fazel Darbandi et al., 2018). However, whether *Tbr1* similarly regulates spontaneous network activity even during early developmental stages remains to be addressed.

**Figure 5:**
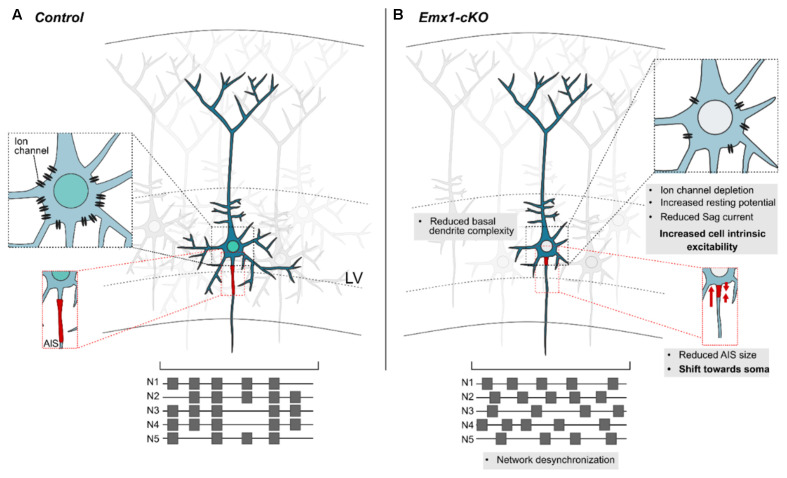
*Nr2f1* controls the bioelectric and cytostructural nature of layer V cortical neurons. **(A)** Schematic representation of a layer V pyramidal neuron in a physiological context. A specific asset of ion channels is displayed on the cell membrane (magnification in the black dashed box) and determines cell electrophysiological properties (i.e., resting membrane potential, sag current, etc.) and proper network synchronization. The firing pattern of five neurons -N1 to N5- is schematized in the lower scheme, in the form of gray squares (representing firing events) distributed along lines (indicating measurement time during recording). Structural features of dendrite complexity and axon initial segment (AIS; magnified in the red dashed box) are also influenced by the cellular bioelectric state. **(B)** Summary of the main defects observed upon loss of *Nr2f1*. Several ion channels are downregulated in mutant neurons (inset in the black dashed box), resulting in increased intrinsic excitability. Furthermore, distinct neurons lose their ability to efficiently fire action potentials in a synchronized way (lower scheme), suggesting that the overall network synchronization is affected. At the morphological level, the complexity of the basal dendrites is drastically diminished upon *Nr2f1* loss, and the AIS is reduced in size and misplaced closer to the soma (magnified in the red dashed box). Modified from Del Pino et al. (2020). LV, layer V.

Recent data have shown that loss of *Nr2f1* from cortical progenitors affects spontaneous network activity, synchronization *in vitro*, as well as intrinsic bioelectric properties of cortical pyramidal neurons *in vivo* (Del Pino et al., 2020)*.* For instance, *Nr2f1* mutant neurons were characterized by increased resting membrane potential, reduced rheobase and sag current, hinting at increased intrinsic excitability (Figure 5). Expression profile analysis revealed that *Nr2f1* transcriptionally regulates a plethora of layer V-expressed ion channels, including the *hyperpolarization-activated cation channel-1* (*HCN1*), a well-known regulator of rhythmic oscillatory activity, ultimately controlling neuronal excitability (Huang et al., 2009; Bonzanni et al., 2018; Marini et al., 2018). Notably, *Nr2f1* can directly bind to *HCN1* regulatory regions *via* several consensus sequences (Del Pino et al., 2020). As a result, both *HCN1* transcript and protein levels are specifically downregulated in layer V neurons upon *Nr2f1* cortical ablation. Interestingly, genetic or pharmacological disruption of HCN channels activity results in dysfunctional somatosensory-motor coordination in mice, such as reduced forelimb reaching accuracy and atypical movements during a single-pellet skill reaching task (Boychuk et al., 2017). These behavioral deficits resemble those displayed in cortical *Nr2f1* c*KO* mice (Tomassy et al., 2010) and remind motor-coordination deficits of *NR2F1* haploinsufficient patients (Bosch et al., 2014; Chen et al., 2016; Rech et al., 2020). Nevertheless, although HCN1 is a potential direct effector of *Nr2f1* in inducing an intrinsic electrophysiological phenotype, other ion channels may contribute to the impairment of network activity and intrinsic excitability upon *Nr2f1* loss. The electric impairment is also accompanied by structural and morphological modifications, in line with previous studies (Tien and Kerschensteiner, 2018). Cortical pyramidal neurons showed reduced complexity of their basal arborization, alongside with a defective axon initial segment (AIS; Del Pino et al., 2020), a specialized region localized between the somatic and axonal compartments, responsible for the initiation of action potentials (Leterrier, 2018). However, since *Nr2f1* also regulates the expression of cytoskeletal genes (Armentano et al., 2006; Alfano et al., 2011), the effect of *Nr2f1* loss on pyramidal neuron morphology and on the AIS size could also depend on direct modulation of cytoskeletal proteins, rather than being an indirect effect of altered excitability. A similar mechanism has been demonstrated for another key determinant of corticogenesis, *autism susceptibility candidate-2* (*AUTS2*; Hori et al., 2014). These studies revealed impairments in bioelectric properties of cortical neuronal populations and/or network activity upon loss of transcriptional regulators, illustrating that genetic determinants implicated in NDDs often link neuronal specification to cellular excitability (Huang and Hsueh, 2015; Rodríguez-Tornos et al., 2016; Khandelwal et al., 2021; Runge et al., 2021). In summary, *Nr2f1* might influence cell excitability, either *via* the regulation of ion channels, or *via* the control of cytoskeletal components shaping neuronal structural features. Altered excitability and/or morphology of layer V neurons in BBSOAS patients might be the cause of pathological features, such as ID, epileptic traits, but also poor motor coordination of fine skilled movements.

## An Intrinsic Role of *Nr2f1* in Interneuron Specification, Migration, and Function

To maintain a proper balance of cortical network activity, different types of inhibitory interneurons will modulate the activity of excitatory pyramidal neurons. Cortical GABAergic interneurons are generated in the ventral telencephalon and sequentially migrate into the dorsal telencephalon to integrate the forming local circuitry. In the ventral telencephalon, *Nr2f1* is first expressed in progenitors in a broad and graded pattern spanning the caudal, medial and lateral ganglionic eminences (CGE, MGE, and LGE). Then, it becomes gradually restricted to the CGE, before being maintained by distinct subpopulations of CGE-derived GABAergic interneurons, such as Vasoactive Intestinal Peptide (VIP) and Calretinin (CR)-expressing interneurons (Flames et al., 2007; Lodato et al., 2011; Touzot et al., 2016). As in rodents, both human *NR2F1* and *NR2F2* are also expressed in embryonic progenitors and migrating interneurons (Reinchisi et al., 2012; Varga et al., 2015; Alzu’Bi et al., 2017), and their expression is maintained in some mature cortical interneuron subtypes, with a preference for CGE-derived interneurons.

The use of a specific conditional *KO* mouse model showed that the loss of *Nr2f1* expression in interneuron precursors affected the balance between MGE- and CGE-derived populations, without altering the overall interneuron number. Specifically, late-born CGE-derived VIP+ and CR+ interneurons were decreased in number and aberrantly migrated towards the forming neocortex. Conversely, the number of early-born MGE-derived Parvalbumin (PV)-expressing interneurons increased, possibly due to augmented proliferation in the MGE (Lodato et al., 2011; Touzot et al., 2016). As a result of the enlarged PV+ inhibitory population, interneuron-specific *cKO* animals appear to be more resistant to seizure induction (Lodato et al., 2011). However, this does not recapitulate the human syndrome, since BBSOAS patients are often affected by epileptic seizures (Chen et al., 2016; Rech et al., 2020). A possible explanation is that in patients, *NR2F1* is simultaneously lost from both dorsal and ventral telencephalon, equally affecting the function of excitatory neurons and inhibitory interneurons and leading to an imbalance in the overall brain activity. Instead, in the interneuron-specific *cKO* animals, only interneurons are affected by *Nr2f1* loss, while the function of pyramidal neurons is not directly altered, hence resulting in a more resilient network state.

Impaired excitation/inhibition (E/I) balance upon loss of *Nr2f1* has been supported by a recent study employing the first “patient-specific” *Nr2f1* mutant mouse model, carrying in heterozygosity the *Nr2f1* point mutation R112K (Zhang et al., 2020). Mutants are characterized by reduced expression of dorsal telencephalic markers and a concomitant increase of ventral ones. This translates, at later stages of development, into a decrease of cortical excitatory pyramidal neurons and an increase of inhibitory interneurons, thus perturbing the overall E/I balance. At the electrophysiological level, this imbalance leads to a frequency reduction of miniaturized excitatory postsynaptic currents and an increase in inhibitory ones. E/I imbalance has been reported as a possible cause for both epilepsy and ASD (Powell, 2013), two pathological features frequently reported in BBSOAS patients. Whether this is the case for patients carrying variants different from the one tested in this study, remains to be assessed. So far, the use of this novel mouse model carrying a human-specific mutation succeeded in correlating E/I imbalance with ASD-like behavior deficits, such as reduced sociability, excessive grooming, repetitive and anxiety-like behaviors (Zhang et al., 2020), but whether such E/I imbalance also underlies BBSOAS-related epilepsy has yet to be directly tested.

## Beyond The Neocortex: *Nr2f1* Regulates The Anatomical and Functional Development of The Hippocampus

All the previously described *Nr2f1*-dependent processes orchestrating neocortical development and network maturation could be directly connected to ID reported in BBSOAS patients (Rech et al., 2020). However, the hippocampus (HP), a key cortical structure regulating cognitive processes such as learning and memory (Broadbent et al., 2004; Kumaran and Maguire, 2005; Hannula et al., 2013) also resulted affected in BBSOAS patients. Due to the central role of the HP in controlling key cognitive skills, even small developmental defects can have a dramatic impact on behavioral performances (Mátéffyová et al., 2006; Ramos, 2008; Wan et al., 2021; Zhao et al., 2021). While the dorsal hippocampal pole is more involved in spatial navigation (Moser et al., 1995), the ventral one controls non-spatial learning and emotional behaviors (Kheirbek et al., 2013; Wang et al., 2013). Interestingly, some BBSOAS patients show hippocampal morphological defects, such as dysmorphic HP or hippocampal malrotation (Cardoso et al., 2009; Chen et al., 2016), suggesting that specific cognitive abnormalities reported in these patients could derive from HP developmental impairments. Cortex-specific conditional mouse models (*cKOs*) and constitutive *HET* animals have helped dissect the *Nr2f1* role in HP morphogenesis and function (Flore et al., 2016; Parisot et al., 2017; Chen et al., 2020), as well as in HP-controlled adult behavior (Tomassy et al., 2010; Flore et al., 2016; Contesse et al., 2019). Loss of *Nr2f1* in the cortex at early stages leads to severe shrinkage of the intermediate and dorsal but not ventral regions of the HP, as well as impaired connectivity of topographic inputs from the entorhinal cortex (Flore et al., 2016). This ultimately results in selective impairment of spatial learning and memory, whereas emotional behavior and visual cue navigation remain intact (Flore et al., 2016). The impact of *Nr2f1* loss on HP development depends on both NP proliferation and neuronal migration during embryonic and early postnatal development (Parisot et al., 2017; Bertacchi et al., 2019b). Differently from the neocortex, *Nr2f1* acts as a pro-mitotic factor during hippocampal and dentate gyrus (DG) development and positively regulates NP migration along the primary, secondary, and tertiary DG matrices (Parisot et al., 2017). Interestingly, several genes are differentially expressed in gradients along the D-V HP axis (Leonardo et al., 2006; Lein et al., 2007; O’Reilly et al., 2015; Lee et al., 2017), but *Nr2f1* is one of the few genes which regionalized expression is directly linked to hippocampal development and behavioral function. The existence of a link between *Nr2f1* graded expression and hippocampal regionalization reminds the one described in the neocortex (Zhou et al., 2001; Armentano et al., 2007; Alfano et al., 2014), indicating that gradient gene expression might be a general mechanism for proper structural brain development.

A recent study used an *Nr2f1* *HET* mouse model to better recapitulate the haploinsufficiency condition of BBSOAS patients (Chen et al., 2020). Electrophysiological investigation in hippocampal slices revealed impairment of two major cellular processes involved in learning and memory: long-term potentiation and long-term depression (Chen et al., 2020), suggesting that altered hippocampal synaptic plasticity may contribute to BBSOAS cognitive impairments. Although *HET* mutants confirmed decreased hippocampal volume, such defect was not as severe as in homozygous *cKO* mutants. Furthermore, normal spatial memory but specific deficits in fear memory were described (Chen et al., 2020). The contrasting results obtained in cortical *cKO* and *HET* animals might be attributed to the different genetic nature and entity of the *loss-of-function* mouse model. For instance, in *cKO* mice, loss of both *Nr2f1* alleles is limited to the cortex, and leads to severe but spatially restricted anatomical defects, accompanied by specific behavioral abnormalities. *Vice versa*, the constitutive *HETs* lack just one allele, but the loss affects the entire organism. This might explain the downsized morphological defects (since approximately 50% of the *Nr2f1* protein pool is produced and functional in *HETs*) as well as the more complex behavioral phenotype (due to the disruption of other brain structures involved in the regulation of learning, memory, and emotional behavior in *HET* animals). Further studies will be necessary to dissect the exact contribution of hippocampal deficits on BBSOAS ID and to distinguish it from malformations due to other cortical or subcortical regions.

## *Nr2f1*-Dependent Development of The Sensorimotor System

The progressive development and refinement of proto-areas and functional areas, implies that distinct cortical regions wire to other cortical and/or subcortical structures to orchestrate complex behaviors, such as planning, control, and execution of voluntary movements. The circuit regulating voluntary movements was first described as the cerebro-cerebellar pathway (Brodal, 2010). However, several recent studies have shown how other brain regions, such as the pontine nuclei and the ventrolateral nuclei of the thalamus (VL), also retain an active role in the regulation of distinct voluntary movements (Schwarz and Thier, 1999; Bosch-Bouju et al., 2013; Guo et al., 2021). This network connects the cerebral neocortex to the cerebellum *via* the pontine nuclei and *vice versa* the cerebellum to the neocortex through the VL nucleus of the thalamus. In humans, up to 90% of the neocortical layer V projections are estimated to target the pontine nuclei, a percentage that is even higher in rodents, where almost the whole layer V population is projecting towards these structures. Similarly, up to 90% of pontine mossy cells are sending their axons to the cerebellum. Once the information reaches the cerebellum, it is further elaborated and a feedback output is sent back to the neocortex, through the VL (Schwarz and Thier, 1999; Bosch-Bouju et al., 2013). In both the neocortex and the cerebellum, neuronal populations involved in the regulation of voluntary movements are arranged in topographically organized maps, with different body parts being represented in largely continuous maps in the somatosensory cortex (Woolsey and Van der Loos, 1970; Welker, 1971; Chapin and Lin, 1984; Fabri and Burton, 1991), and discontinuous, fractured maps in the cerebellum (Bower et al., 1981; Bower and Kassel, 1990; Bower, 2011). The intercalated regions of this network receive and integrate signals from different structures, thus helping to coordinate and seamlessly execute fine motor behaviors (Badura et al., 2013; Mottolese et al., 2013). Cortical descending tracts, such as the cortico-spinal tract then convey the motor command to the spinal cord.

BBSOAS patients often present motor abnormalities, such as delayed motor development, poor motor planning and coordination, as well as stereotyped repetitive movements (Rech et al., 2020). A detailed patient report hypothesized that some of these motor impairments might be traced back to defects of the voluntary movement network (Bojanek et al., 2020). In addition, defects in both reaction time and movement accuracy could specifically arise from abnormal functioning of the cerebro-ponto-cerebellar pathway (Bojanek et al., 2020). Studies on the motor functions of a bigger BBSOAS cohort would help to further characterize the voluntary movement network affected by *NR2F1* haploinsufficiency.

**Figure 6:**
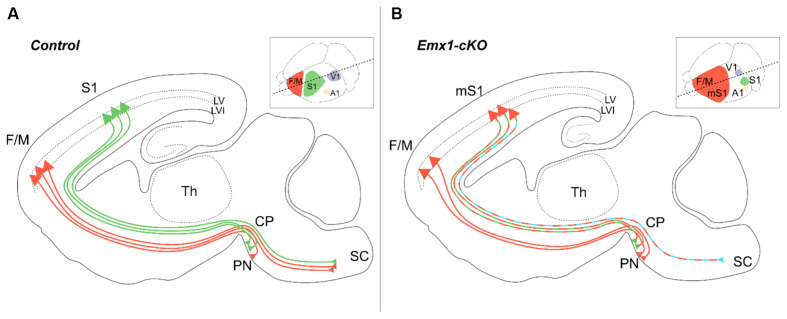
Layer V pyramidal neuron subtype specification is under the control of *Nr2f1* cortical expression. In *wild-type* mice **(A)**, subcortical projecting neurons of layer V target distinct brain regions depending on their area localization. Layer V neurons from the fronto-motor areas (F/M, orange) mainly project to the spinal cord (SC) with very few axons targeting the pontine nuclei (PN), whereas layer V neurons from S1 (green) mainly target the pontine nuclei with fewer projections towards the SC. In conditional cortical-specific *Nr2f1* mutants (*Emx1-cKO*) **(B)**, F/M axons are incorrectly wired and stop at the level of the cerebral peduncle (CP), resulting in a depletion of corticospinal connections upon *Nr2f1* loss. Layer V axons from the motorized S1 (mS1) still project to the PN (green/orange dashed lines), but not to the SC, which is instead aberrantly innervated by layer VI mS1 neurons (blue/orange dashed line). A1, primary auditory area; CP, cerebral peduncle; F/M, fronto-motor area; LV, layer V; LVI, layer VI; mS1, motorized primary somatosensory area; PN, pontine nucleus; S1, primary somatosensory area; SC, spinal cord; V1, primary visual area.

Previous reports in mice have helped describe the development of cortical descending tracts and started unraveling the specific assets of molecular players, including axon guidance cues, signaling molecules, cell adhesion proteins as well as transcription factors necessary for the assembly of the voluntary movement circuitry in general, and the development of the corticospinal tract in particular (Welniarz et al., 2017). *Nr2f1* mutants have been proved efficient models for recapitulating some motor impairments that may underlie BBSOAS-like motor deficits. The impairment observed upon loss of *Nr2f1* function in the tangential (area) organization of the forming neocortex is also accompanied by radial (laminar) changes in the structure of neocortical layers, which ultimately results in abnormal connectivity between the cortex and its subcortical targets (Armentano et al., 2007; Tomassy et al., 2010; Greig et al., 2013; Alfano et al., 2014). In physiological conditions, subcortical projection neurons from M1 and S1 follow slightly different paths: while M1 axons mainly reach the spinal cord and constitute only a small fraction of the pontine nuclei innervation, S1 axons almost exclusively target the pontine nuclei (Figure 6A). However, following the disruption of cortical area specification caused by *Nr2f1* inactivation (Zhou et al., 2001; Armentano et al., 2007; Alfano et al., 2014), these proportions change with both S1 and M1 populations equally projecting to the pontine nuclei. Only a depleted population of axons coming from the motor-like sensory cortex (“mS1”) will reach the spinal cord in mutants (Figure 6B; Tomassy et al., 2010). This defective connectivity has, most probably, a deleterious effect on motor execution and behavior. *Nr2f1* *cKO* animals are characterized by abnormal dexterity and voluntary movement execution, in terms of reduced forelimb reaching accuracy and atypical movements during a single-pellet reaching task, as well as hyperactive features (Tomassy et al., 2010; Contesse et al., 2019). These observations support the *cKO* mice as a reliable animal model for further understanding the impaired development and/or defective circuits at the origin of the voluntary movement execution and hyperactive behavior. Further, a recent study assessing the role of pontine nuclei in voluntary movements supports their direct involvement in the timing and accuracy of movements rather than its initiation. Specifically, upon optogenetic disruption of the pontine nuclei activity, the animals retained the ability to initiate the reaching phase of the single-pellet task but showed either reduced precision of the grasping phase or a general delay in the complete execution of the task (Guo et al., 2021). Since the defects showed by *Nr2f1* cortical* cKO* mice resemble those presented in this work, with an almost intact reaching phase but inaccurate grasping (Tomassy et al., 2010), it is tempting to speculate that gradient cortical *Nr2f1* expression could impart topographic information to descending tracts during development by influencing their connectivity to subcortical structures, such as the pontine nuclei and the spinal cord. This would imply that *Nr2f1* can link key processes of corticogenesis, such as area identity, layer formation and tract connectivity, allowing the correct development of voluntary motor networks.

## More than Meets The Eye: *Nr2f1* Orchestrates Visual System Development from Peripheral Retinal to Central Thalamic and Neocortical Areas

**Figure 7:**
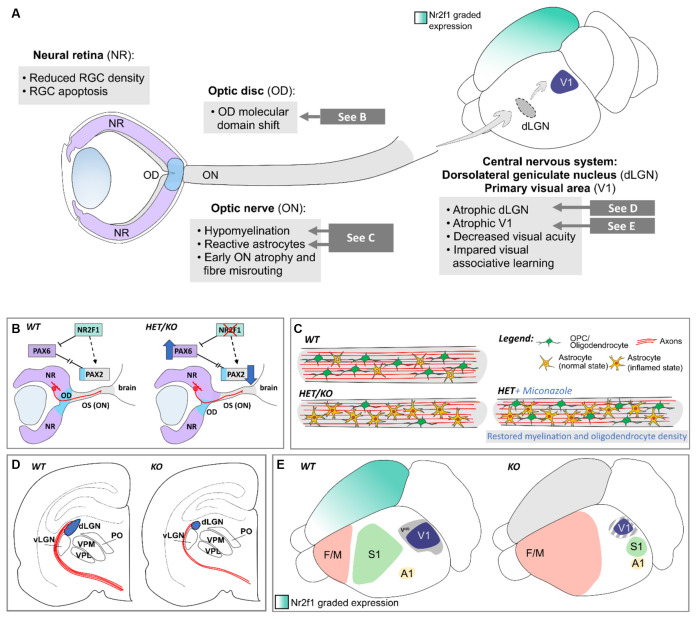
*Nr2f1*-dependent development of the peripheral and central visual system in mouse. **(A)** Overview of the phenotypes observed in several structures of the mouse visual system upon *Nr2f1* loss, comprising the neural retina (NR), the optic disc (OD), the optic nerve (ON), and distinct central structures, such as the dorsolateral geniculate nucleus (dLGN) and the primary visual area (V1). **(B)** *Nr2f1* loss causes a molecular shift of the Pax6+ NR domain towards the Pax2+ optic stalk (OS)/ON domain, resulting in aberrant positioning of the OD border and axonal misguidance of retinal ganglion cell (RGC) axons exiting the developing eyeball. **(C)** *Nr2f1* haploinsufficient (*HET*) or depleted (*KO*) mice show decreased size of the ON compared to wild-type (*WT*) animals, resembling ON atrophy reported in BBSOAS patients. Upon *Nr2f1* loss, a population of Sox2+ astrocytes with inflamed morphology outnumbers oligodendrocyte progenitors (OPCs), resulting in ON inflammation and hypomyelination. While the myelination defect can be rescued by Miconazole treatment, the high proportion of reactive astrocytes and consequent gliosis and the general ON atrophy are not reverted by pro-myelinating chemical drugs. **(D)** Reduced axonal innervation and connectivity (in red) from the NR to the atrophic dLGN (in blue) in *Nr2f1*-deficient mice. **(E)** Atrophic V1 (blue) and impaired maturation of secondary associative visual cortices (visual high order cortex -V^HO^-; gray) due to arealization defects upon *Nr2f1* loss. A1, primary auditory area; dLGN, dorsolateral geniculate nucleus; F/M, frontal/motor area; NR, neural retina; OD, optic disc; ON, optic nerve; OPC, oligodendrocyte precursor cell; OS, optic stalk; S1, primary somatosensory area; V1, primary visual area; V^HO^, high-order visual associative secondary areas; vLGN, ventrolateral geniculate nucleus.

BBSOAS patients were first identified for their unique combination of cerebral and visual impairments, suggesting that besides cortical development, NR2F1 might also influence the establishment of the visual system. Several clinical reports describe that a major clinical feature of BBSOAS patients is the profound impairment of visual performances (Bosch et al., 2014; Chen et al., 2016; Kaiwar et al., 2017; Rech et al., 2020; Starosta et al., 2020; Zou et al., 2020), due to optic nerve atrophy and/or optic nerve hypoplasia, decreased visual acuity and cerebral visual impairment, defined as impaired analysis and interpretation of visual stimuli (Bosch et al., 2016). While recent reports are detailing the ophthalmological features of visually impaired BBSOAS children (Zou et al., 2020; Jurkute et al., 2021), mouse models offer again the unique opportunity to further understand the role of *Nr2f1* in vision, from the developmental and the functional points of view. Previous *Nr2f1* conditional mouse models failed in reproducing key patient eye defects, such as malformed optic discs and optic atrophy (Tang et al., 2010), but constitutive models, better mimicking patients’ haploinsufficiency, efficiently recapitulate a plethora of BBSOAS-like symptoms (Bertacchi et al., 2019a). From the peripheral-most relay points of the visual system, i.e., the retina and the optic nerve, to more central brain structures deputed to the analysis of visual stimuli, such as the visual thalamus and cortices, we summarize in this section how *Nr2f1/NR2F1* graded expression in distinct relay-points of the developing visual system impacts the function of mouse and human vision (Figure 7A).

## Impaired Retinogenesis and Non-Progressive Decrease of Rgc Density upon *Nr2f1* Loss

Optic nerve atrophy (ONA) and/or optic nerve hypoplasia (ONH), often reported in BBSOAS patients (Rech et al., 2020), are characterized by a reduced optic nerve (ON) exiting the retina, together with thinning of retinal layers, retinal ganglion cell (RGC) and retinal nerve fiber layers (RNFL), where RGCs and their axons reside, respectively (Jurkute et al., 2021). *NR2F1* is dynamically expressed in different retinal cell types of the human eye, including RGCs (Bertacchi et al., 2019a), which form the ON by elongating their axons from the retina to the brain. The high degree of conservation of *Nr2f1* expression between the human and rodent visual pathway (Alfano et al., 2014; Bertacchi et al., 2019b) allowed the use of the mouse as a model system to study BBSOAS-related visual impairments. Starting from the peripheral-most structure of the visual system, the retina, *Nr2f1* shows a dynamic expression along a dorsal-low to ventral-high gradient, whereas its homolog *Nr2f2* displays an opposite gradient (Tang et al., 2010, 2015). This dorso-ventral (D-V) *Nr2f1* gradient has specific consequences on neural development: D-V specification of regional retinal identities is compromised in the absence of both *Nr2f* genes, with Pax6 expression being abnormally enhanced at the expense of key genes imparting a ventral identity to the mouse eye, such as *Ventral anterior homeobox-1* and *-2* (*Vax1* and *Vax2*, respectively; Barbieri et al., 1999; Mui et al., 2005; Tang et al., 2010; Figure 7B). Later in development, *Nr2f1* and *Nr2f2* convey the D-V regional information to direct the expression of Opsins, a family of light-sensitive proteins localized in photoreceptors (Satoh et al., 2009). The presence of *Nr2f* D-V gradients also suggests that these genes might be involved in the appropriate formation of the retinotopic projection map of RGC axons, the only long-range projections from the retina to the brain. Even if further experiments will be necessary to tackle this question, recent data about axonal misrouting in *Nr2f1*-deficient mice point in this direction (Jurkute et al., 2021). Besides axonal guidance, the number of RGCs in the retina was also affected in mutants, since a stable decrease in RGC density was reported in adult *HET* mice (Jurkute et al., 2021). This defect originates early in development and possibly involves RGC apoptosis around birth (Bertacchi et al., 2019a). Notably, the RGC decrease found in the mouse model fits well with the finding of ONA and ONH in patients, displaying a decreased amount of RGC fibers in the ON and showing non-progressive, stable reduction of RGC and RNFL thickness. Overall, these observations suggest an early developmental cause for the axonal depletion within the ON (Bosch et al., 2014; Chen et al., 2016; Jurkute et al., 2021).

Finally, little is known whether *Nr2f1* can influence the development and distribution of other retinal cell types. Given that *Nr2f1* expression is maintained in the ganglion cell and inner nuclear layers and, at lower levels, in the outer nuclear layer of the adult mouse retina (Inoue et al., 2010; Bertacchi et al., 2019a; Jurkute et al., 2021), it is reasonable to hypothesize a role for Nr2f1/NR2F1 in the maintenance and functionality of mature retinal cells. High *Nr2f1* expression was also detected in both mouse and human retinal pigment epithelial (RPE) cells (Jurkute et al., 2021); it is tempting to speculate that *Nr2f1* could then influence photoreceptor function indirectly *via* RPE-mediated mechanisms.

## *Nr2f1* Places The Optic Disc Boundary by Regulating Neural Retina and Optic Stalk Genes

Early studies unraveled an *Nr2f*-modulated network of genes expressed in the neural retina and optic stalks, such as *Pax6*, *Pax2*, *Otx2* and *melanocyte inducing transcription factor* (*Mitf*), necessary for eye development and for placing the optic disc region between the neural retina and the optic stalk (Figure 7B; Schwarz et al., 2000; Tang et al., 2010; Pattabiraman et al., 2014). The optic disc, specified by the combinatorial expression of transcription factors, is in turn fundamental for producing signaling molecules for RGC axonal guidance (Deiner et al., 1997). While neither *Nr2f1* nor *Nr2f2* single *KO* mice seemed to develop major optic disc abnormalities, double *KO* mice (characterized by the combined inactivation of both homologs) showed severe coloboma, microphthalmia, and misplacement of the eye border resulting in a proximal shift of the optic disc (Tang et al., 2010). The compensatory effect of both homologs (*Nr2f1* and *Nr2f2*) during mouse eye development was quite surprising since BBSOAS patients show ocular impairments with high penetrance, already upon loss of one single copy of *NR2F1*. The discrepancy was probably due to the mouse model initially used to dissect the role of *Nr2f1* in eye development, a conditional mutant in which *Nr2f1* expression was selectively abolished in retinal tissue (Furukawa et al., 1997; Swindell et al., 2006; Tang et al., 2010). The use of an *Nr2f1* constitutive mutant instead, characterized by a reduction in *Nr2f1* dosage in the entire organism and from the earliest stages of development, allowed a better reproduction of BBSOAS-like conditions, and helped demonstrate how this genetic network is disrupted by the loss of *Nr2f1* alone (Bertacchi et al., 2019a). In fact, the sole absence of *Nr2f1* is sufficient to cause a shift of the border between the neural retina and the optic stalk, which in turn has heavy consequences on the expression of optic disc molecular determinants, such as Netrin1, and on the RGC projections exiting the eyeball (Jurkute et al., 2021; Figure 7B). However, compared to *KO* mutants, *Nr2f1* *HET* animals have more subtle defects, that are partially recovered during late embryonic development (Bertacchi et al., 2019a). This is in striking contrast to BBSOAS patients, that continue to show various eye malformations, such as excavated and pale optic discs from childhood to adulthood. Understanding the origin for these species-specific differences will require further studies.

## Astrogliosis and Hypomyelination Converge to Optic Nerve Atrophy in A *Nr2f1*-Deficient Model

Besides showing atrophy, the ON of the mouse BBSOAS model revealed low levels of myelination (Bertacchi et al., 2019a), caused by a delay in oligodendrocyte precursor proliferation and differentiation (Figure 7C). In normal physiological conditions, mouse oligodendrocytes are generated in the pre-optic area, then enter the ON guided by local signaling molecules, proliferate *in loco* and finally differentiate in the first post-natal month into mature oligodendrocytes, wrapping RGC axons to allow optimal signal conductivity (Tsai and Miller, 2002; Stolt et al., 2004; Ono et al., 2017). These processes are impaired in *HET* mice and almost absent in *KOs*, suggesting a key role for *Nr2f1* in the maturation of oligodendrocytes and in the myelination process, consistently with a previous report (Yamaguchi et al., 2004). Hypomyelination could in turn exacerbate the loss of ON axonal fibers by affecting RGC survival (Teixeira et al., 2016). It would be interesting to investigate whether this is a common mechanism happening in other structures, for example in the neocortical white matter, since MRI scans have revealed impaired myelination in some BBSOAS patients (Chen et al., 2016; Rech et al., 2020). In parallel to hypomyelination, the atrophic ON in mutant mice is further impacted by inflammatory processes reactivating dormant astrocytes (Bertacchi et al., 2019a), as observed by both morphological changes and expression of inflammatory markers such as *SRY-box transcription factor-2 (Sox2)* (Figure 7C; Bani-Yaghoub et al., 2006; Hernandez et al., 2008; Zhang et al., 2013; Pekny et al., 2014). Hence, *Nr2f1* represents the core of a genetic network regulating the astrocytic inflammatory process and neuron-astroglia cell fate decision in other brain regions, such as the adult mouse hippocampal neurogenic niche (Bonzano et al., 2018). Further studies will be necessary to unravel the temporal order and possible causative relationships between these distinct processes—oligodendrocyte hypomyelination, astrocyte inflammation, and RGC survival–that by influencing each other could exacerbate the *Nr2f1*-dependent phenotype.

Likely due to both inflammation and ON hypomyelination, electrophysiological recordings disclosed a significant delay in the conduction velocity of visual stimuli along the ONs of mutant animals (Bertacchi et al., 2019a). Interestingly, myelination could be artificially boosted *in vivo* by early treatments of specific chemical drugs, in both physiological conditions and pathological models (Harlow et al., 2015; Najm et al., 2015; Porcu et al., 2015; Eleuteri et al., 2017; Su et al., 2018). Early post-natal injection of Miconazole, a chemical drug promoting oligodendrocyte differentiation, efficiently rescued the hypomyelination phenotype of *Nr2f1* haploinsufficient mice (Figure 7C; Bertacchi et al., 2019a), opening promising therapeutic avenues for BBSOAS visually-impaired patients and, more in general, supporting the feasibility of employing mouse models for therapeutic drug screening. However, Miconazole treatment rescued myelination but had no effect on astrogliosis, indicating that the two pathological events are independent in BBSOAS-like optic neuropathy and that additional treatments should be tested to revert the ON inflammatory state.

## *Nr2f1* Influences Complex Visual Associative Behaviors by Controlling Thalamic and Neocortical Development

Abnormal perception, elaboration, and interpretation of visual stimuli occurring in patients with cerebral visual impairments (affecting around 70% of BBSOAS patients; Bosch et al., 2016; Bertacchi et al., 2019a; Rech et al., 2020) suggests an impairment of higher-order visual centers (such as primary and associative visual cortices) besides peripheral structures, such as the retina and the ON (Philip and Dutton, 2014). Even in this context, mouse models offer the unique opportunity to investigate the impact of *Nr2f1* loss on the establishment of central thalamic and neocortical structures and to characterize the electrophysiological and behavioral consequences. Mouse *Nr2f1* is dynamically expressed in the thalamus, with high levels in the dorso-lateral geniculate nucleus (dLGN) receiving ON fibers (Qiu et al., 1994; Armentano et al., 2006; Alzu’Bi et al., 2017). Upon *Nr2f1* loss, the thalamic nuclei are affected in their size and connections, in turn impinging on the maturation of primary and secondary visual areas (Figures 7D,E; Zhou et al., 2001; Armentano et al., 2007; Chou et al., 2013). Interestingly, other key visual developmental factors, such as *Sox2*, can converge to similar thalamic phenotypes, when lost or mutated, by affecting the size and connection of the dLGN in a similar way (Mercurio et al., 2021). This suggests that *Nr2f1* might belong to a complex genetic network that is fundamental for the correct establishment of thalamic nuclei and their wiring to both cortex and retina. Electrophysiological recordings of visually evoked potentials in the dLGN of *Nr2f1* *HET* mice revealed delayed transmission of visual stimuli and decreased visual acuity compared to *wild-type* littermates, consistently with low visual acuity (Bosch et al., 2014; Chen et al., 2016; Rech et al., 2020) and non-degenerative vision loss in BBSOAS patients (Jurkute et al., 2021).

Finally, the cerebral visual impairment described in patients could depend on the aberrant elaboration of visual stimuli in higher-order associative cortices (Malkowicz et al., 2006; Philip and Dutton, 2014; Bosch et al., 2016). As mentioned above, *Nr2f1* expression in thalamic and cortical structures is essential for the activity-dependent refinement of the secondary visual cortex in mouse (Figure 7E; Chou et al., 2013), implying that *Nr2f1* could control the maturation of higher-order associative cortices. Hence, the establishment of visual associative behavior was evaluated in *Nr2f1* *HET* and control mice *via* a light-dependent operant conditioning task, in which a reward was obtained only when a visual stimulus (a small light bulb) was present and switched on. While control animals learned to associate the dim visual stimulus with the operant task, mutant animals failed to do so, somehow recapitulating an associative deficit in the interpretation of visual stimuli (Bertacchi et al., 2019a). However, *Nr2f1*-deficient mice were still able to learn and execute complex tasks (Flore et al., 2016; Bertacchi et al., 2019a), suggesting a specific impairment in the perception and elaboration of visual stimuli in high-order cortices rather than a generalized defect during the learning process. The visual impairments at the cortical level due to reduced *Nr2f1* dosage could contribute to one of the main features of ID reported in BBSOAS children.

## Conclusions and Future Directions

Because of their highly interactive nature and their involvement in several simultaneous processes, early expressed transcriptional regulators have always constituted a difficult subject when approached to dissect their molecular mechanisms of action. The nuclear receptor *Nr2f1* makes no exception, as it regulates several cellular programs, sometimes bearing opposite effects in different regions and even at different developmental stages. A possible explanation for such functional differences could reside in the tissue- and time-specific availability of distinct assets of co-factors, through which *Nr2f1* enforces transcriptional regulation of target genes. So far, very few molecular interactors have been reported and, in most cases, it is still difficult to understand whether single or both *Nr2fs* are part of the same regulatory network. Moreover, most studies on *Nr2f* molecular interaction and transcriptional regulation were carried out *in vitro*, where the availability of Nr2f proteins and of their presumptive interacting factors is not closely mimicking physiological conditions (Cooney et al., 1992; Kliewer et al., 1992; Tran et al., 1992). To complement these studies, genome-wide expression analysis of *Nr2f1*-expressing cell populations isolated from distinct brain regions and at different developmental stages is required. This would help to more precisely characterize *Nr2f1*-regulated transcriptional mechanisms and to identify novel targets, in a time- and region-specific manner. Furthermore, mass spectrometry on similar samples would give insights into the identity of elusive co-factors involved in the various regulatory machineries in discrete developmental contexts and brain areas. The use of more physiological systems, such as living human cells, could also improve our understanding of *NR2F1* molecular functioning.

Taking advantage of the high evolutionary conservation of Nr2f proteins among different species, several models have been used in the past to investigate their function *in vivo*. In *D. melanogaster*, for example, the *Nr2f* ortholog seven up (*SVP*) is a key factor for cell identity acquisition in both the central nervous system and the developing eye (Begemann et al., 1995; Kanai et al., 2005; Benito-Sipos et al., 2011). Similarly, the *C. elegans* ortholog UNC55 acts in cell fate determination, enabling the differentiation of two discrete populations of motor neurons (Walthall and Plunkett, 1995; Mimi Zhou and Walthall, 1998; Petersen et al., 2011). Finally, in *X. laevis, xCOUP-TFA* and *B* direct the antero-posterior patterning of the central nervous system (Van Der Wees et al., 1996; Tanibe et al., 2012).

To date, the most employed animal model is by far the mouse, and several different mutant lines have been generated in the attempt to dissect the wide-ranging function of *Nr2f1* during brain development (Table 1). Due to the *Nr2f1* pleiotropic nature, the analysis performed in different models occasionally produced contradictory results. We discussed, for example, how a retina-specific conditional *Nr2f1* *cKO* model is not efficiently reproducing the BBSOAS-like eye development deficits (Tang et al., 2010), as it is instead reported in *Nr2f1* *HET* mutants (Bertacchi et al., 2019a). In the latter, the loss of function is not limited to a specific compartment but affects the entire developing organism from very early stages, as in human patients (Bertacchi et al., 2019a). Similarly, a ventral telencephalon *Nr2f*1 c*KO*, which specifically affects the generation of interneurons, did not induce a cortical E/I imbalance, in contrast to the appearance of epileptic episodes in BBSOAS patients and electric dysfunctions observed in a mouse model carrying a human-like mutation in heterozygosity (Lodato et al., 2011; Touzot et al., 2016; Zhang et al., 2020).

**Table 1:** List of available Nr2f1 mouse models and main related studies.

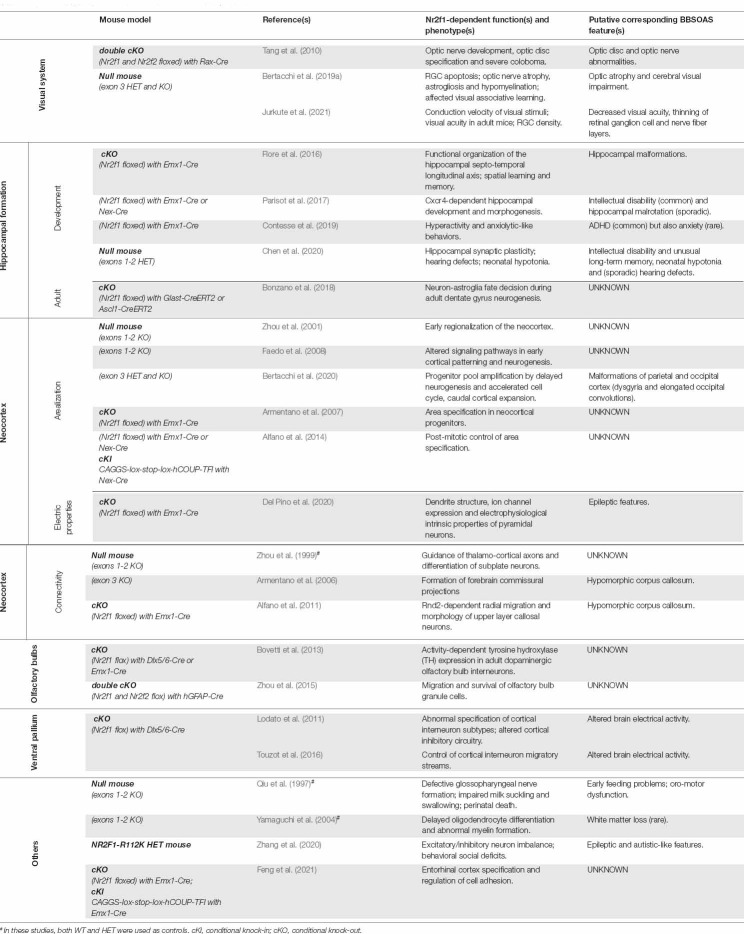

Although not being appropriate for studying the BBSOA Syndrome as a whole, conditional *KO* models are valuable tools to dissect the molecular contribution of *Nr2f1* in distinct brain regions and/or different developmental windows. So far, studies employing *cKO* models helped dissecting the involvement of *Nr2f1* during: (i) neocortical arealization, discriminating between early and late functions in progenitor cells and postmitotic neurons (Armentano et al., 2007; Alfano et al., 2014); (ii) generation of specific classes of interneurons from distinct regions of the ganglionic eminences (Lodato et al., 2011; Touzot et al., 2016); (iii) optic disc and retina formation (Tang et al., 2010); and (iv) formation of higher-order cortical visual areas (Chou et al., 2013), among others.

To further dissect the role of Nr2f1 in specific sub-domains during development, it would be interesting to employ *Nr2f1* neocortical layer-specific *cKO*. This would allow the investigation of cell-intrinsic functions of *Nr2f1* in cell maturation and differentiation in different subpopulations, without affecting the early arealization and lamination processes *in toto*. To this purpose, several layer-specific* Cre* mouse lines could be used to assess *Nr2f1* loss either in layer V (Gong et al., 2007; Taniguchi et al., 2011) or layer IV (Liao and Xu, 2008; Abraira et al., 2017). This would allow to evaluate whether the functional (Del Pino et al., 2020) and/or morphological defects (Hou et al., 2019; Del Pino et al., 2020) observed upon removal of *Nr2f1* from the entire pool of cortical progenitor cells are in fact the result of cell-intrinsic mechanisms, or rather a secondary effect of aberrant area and layer formation.

*Vice versa*, constitutive *HET* mice better recapitulate the complexity and the broad spectrum of developmental defects found in BBSOAS patients. In addition to the use of *HET* models, *constitutive KO* animals (*null*) have been used to further stress the developmental processes and efficiently identify underlying alterations. However, this condition is never observed in human patients, possibly due to foetal or perinatal death. Despite being very accurate in reproducing the symptoms observed in haploinsufficient patients, the *HET* model is still not representative of the entire patient cohort, in which various types of mutations have been identified (spanning from point missense mutations to whole-gene deletions), often corresponding to different degrees of symptom severity. Moreover, genotype-phenotype correlation assessment in human patients shows that whole-gene deletions do not usually lead to the most severe symptoms, hence the *HET* mutant phenotype could be too mild to fully recapitulate the whole BBSOAS spectrum.

A recently developed mouse model, carrying a single copy of a patient-specific mutation (Zhang et al., 2020), seems to be a promising approach, in terms of accuracy and reliability in comparing animal models to BBSOAS patients. It becomes imperative to generate more patient-specific mouse lines and to compare them at the molecular and functional levels, aiming for a better characterization of the genotype-phenotype correlation. However, these models come with their own limitations. For instance, the lissencephalic mouse brain is not suitable for studies on cortical gyrification defects, and the limited pool of murine basal RG cells makes it impossible to study the effects of *Nr2f1* loss on this key population of progenitors. Further, some aspects of cell physiology and biology vary substantially among different species, hence requiring a more human-like model. For this reason, it is necessary to move into *in vitro* human systems, such as 3D brain organoids. The use of brain organoids, coupled with advanced tools of genome editing, such as CRISPR-Cas9, could answer those questions that cannot be investigated in mice. On the other side, being an artificial *in vitro* system, brain organoids come with their own caveats and limitations, such as the lack of cytoarchitecturally-defined layers and areas, and a time-restricted window of investigation, due to their relatively short viability compared to living organisms. In this sense, the combination of *in vivo* animal models and *in vitro* human organoids, each providing its own technical and biological advantages, could be a winning strategy.

In parallel to the studies on animal models, clinical research is advancing our knowledge of BBSOAS features, expanding the list of symptoms as new patients are identified. To date, some aspects of BBSOAS patients are yet to be characterized, and very little is known about their pathological causes. For example, cortical morphology defects have been observed *via* MRI analysis on a small cohort of patients (Bertacchi et al., 2020), and animal models are shown to partially recapitulate such malformations. However, further investigations are required to understand whether this is a shared feature in other patients and evaluate the overall prevalence of these morphological alterations among the whole BBSOAS cohort. Moreover, BBSOAS patients were described to suffer from complex IV deficiency of the mitochondrial respiratory chain (Martín-Hernández et al., 2018), but the overall prevalence of mitochondrial deficits in BBSOAS patients is unknown to date. If present in other patients, it would be interesting to understand whether mitochondrial defects lead to ON atrophy, further impacting RGC physiology (Bertacchi et al., 2019a). Additional investigation using *in vitro* models, such as 2D and 3D cultures of neuronal progenitors, could help elucidate the role of *Nr2f1* in regulating the physiological mechanisms of mitochondrial function, while *in vivo* animal models could help challenge the influence of *Nr2f1* on the general energetic balance of the organism, as a whole.

Overall, the use of experimental models to characterize human diseases, BBSOAS included, is a bidirectional approach. On one hand, human clinical data steer the direction of experimental studies: new patients are identified and reported, new symptoms emerge, and the underlying mechanisms can be investigated in different experimental models. Considering how each model has its both advantages and disadvantages, only their combination will allow a comprehensive and reliable characterization of the pathology. On the other hand, information obtained in experimental models can direct future clinical explorations and be eventually used by clinicians to reveal novel BBSOAS features linked to *NR2F1* haploinsufficiency. Thus, close interactions between *Nr2f1* studies in animal models and clinical assessment of BBSOAS patients constitute the best approach for advancing our understanding of BBSOAS pathophysiology. Finally, as distinct NDDs have been shown to share common pathological features, a better characterization of BBSOAS causative mechanisms will also benefit the understanding of other NDDs, leading to improved diagnosis and development of personalized therapies for a diversified plethora of patients.

## Author Contributions

CT and MB contributed equally to the writing and figures of this review. MS revised the text and figures. All authors contributed to the article and approved the submitted version.

## Conflict of Interest

The authors declare that the research was conducted in the absence of any commercial or financial relationships that could be construed as a potential conflict of interest.

## Publisher’s Note

All claims expressed in this article are solely those of the authors and do not necessarily represent those of their affiliated organizations, or those of the publisher, the editors and the reviewers. Any product that may be evaluated in this article, or claim that may be made by its manufacturer, is not guaranteed or endorsed by the publisher.
